# Cost-effectiveness of prenatal screening and diagnostic strategies for Down syndrome: A microsimulation modeling analysis

**DOI:** 10.1371/journal.pone.0225281

**Published:** 2019-12-04

**Authors:** Wei Zhang, Tima Mohammadi, Julie Sou, Aslam H. Anis

**Affiliations:** 1 Centre for Health Evaluation and Outcome Sciences, St. Paul’s Hospital, Vancouver, British Columbia, Canada; 2 School of Population and Public Health, University of British Columbia, Vancouver, British Columbia, Canada; Illumina Inc, UNITED STATES

## Abstract

**Objectives:**

Down syndrome (DS) is the most frequently occurring fetal chromosomal abnormality and different prenatal screening strategies are used for determining risk of DS worldwide. New non-invasive prenatal testing (NIPT), which uses cell-free fetal DNA in maternal blood can provide benefits due to its higher sensitivity and specificity in comparison to conventional screening tests. This study aimed to assess the cost-effectiveness of using population-level NIPT in fetal aneuploidy screening for DS.

**Methods:**

We developed a microsimulation decision-analytic model to perform a probabilistic cost-effectiveness analysis (CEA) of prenatal screening and diagnostic strategies for DS. The model followed individual simulated pregnant women through the pregnancy pathway. The comparators were serum-only screening, contingent NIPT (i.e., NIPT as a second-tier screening test) and universal NIPT (i.e., NIPT as a first-tier screening test). To address uncertainty around the model parameters, the expected values of costs and quality-adjusted life-years (QALYs) in the base case and all scenario analyses were obtained through probabilistic analysis from a Monte Carlo simulation.

**Results:**

Base case and scenario analyses were conducted by repeating the micro-simulation 1,000 times for a sample of 45,605 pregnant women per the population of British Columbia, Canada (N = 4.8 million). Preliminary results of the sequential CEAs showed that contingent NIPT was a dominant strategy compared to serum-only screening. Compared with contingent NIPT, universal NIPT at the current test price was not cost-effective with an incremental cost-effectiveness ratio over $100,000/QALY. Contingent NIPT also had the lowest cost per DS case detected among these three strategies.

**Conclusion:**

Including NIPT in existing prenatal screening for DS is shown to be beneficial over conventional testing. However, at current prices, implementation of NIPT as a second-tier screening test is more cost-effective than deploying it as a universal test.

## Introduction

Prenatal screening for Down syndrome (DS) (trisomy 21) and other chromosomal abnormalities such as trisomy 13 and 18 are offered in many countries worldwide [1,2]. DS is the most common chromosomal abnormality and the risk of an affected fetus increases with maternal age. DS is associated with intellectual disability and a higher likelihood of other health problems such as congenital heart disease [3–5]. Prospective parents are provided with information about DS, other chromosomal abnormalities and prenatal screening, and make an informed choice regarding whether to have the screening.

Different prenatal screening options are available and include various maternal serum biomarkers and ultrasound measurement of nuchal translucency (NT). Positive screens are referred for diagnostic testing by amniocentesis or chorionic villus sampling (CVS) [6–9]. In recent years, non-invasive prenatal testing (NIPT), a relatively new blood test that measures the amount of cell-free fetal DNA circulating in maternal serum, has been demonstrated to have a greater sensitivity (>99%) and specificity (≥99%) for DS compared to conventional screening tests [10–13]. Due to improved test performance, NIPT greatly reduces the number of false positives that need to be confirmed by invasive tests and thus avoids the potential for procedure-related fetal loss.

In practice, NIPT can be offered either as a first-tier screening test for all pregnant women in a universal NIPT strategy or as a second-tier test for those with positive results from the conventional screening tests in a contingent NIPT strategy. Although NIPT is effective in detecting DS, according to a systematic review by García-Pérez et al. [14] the cost-effectiveness of contingent NIPT is still uncertain while universal NIPT is largely not cost-effective. However, all 12 studies identified in the review used the number of DS cases detected as the effectiveness measure instead of quality-adjusted life years (QALYs), the more commonly used effectiveness measure. Some previous studies have estimated utility weights for the outcomes of pregnancy and prenatal screening (e.g., procedure-related miscarriage and DS-affected births) [15–17], which could be used to calculate QALYs. Also, most of the previous cost-effectiveness analyses (CEAs) used cohort-based decision analytic models without considering heterogeneity and tracking of individual histories.

To address limitations in existing cost-effectiveness studies, the objective of this study was to assess the cost-effectiveness of contingent and universal NIPT strategies at the population level using a microsimulation decision-analytic model from the perspective of the publicly funded health care payer in British Columbia (BC), Canada.

## Materials and methods

### Model overview and strategy comparisons

**Fig 1:**
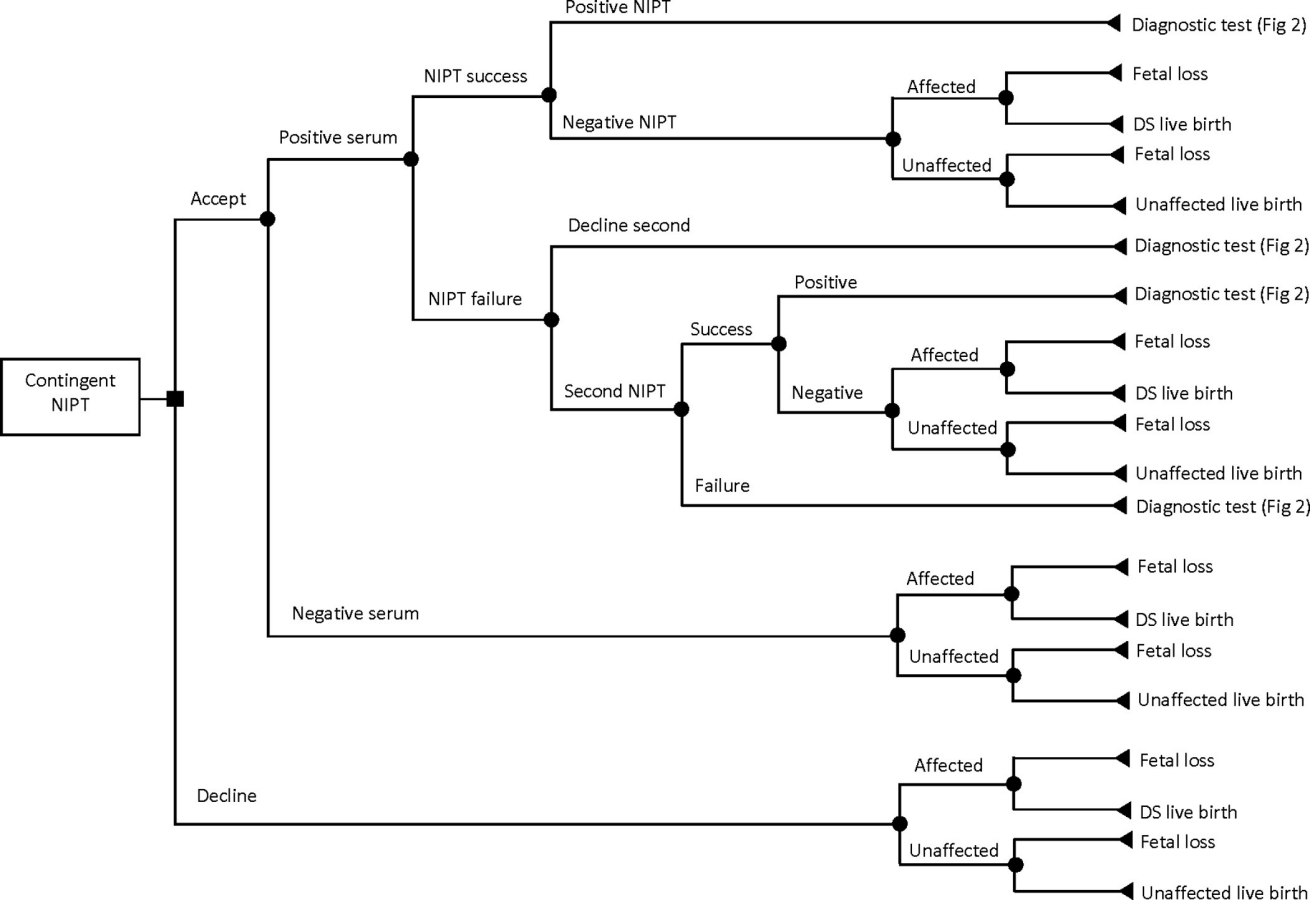
Pathway for contingent non-invasive prenatal testing strategy.

**Fig 2:**
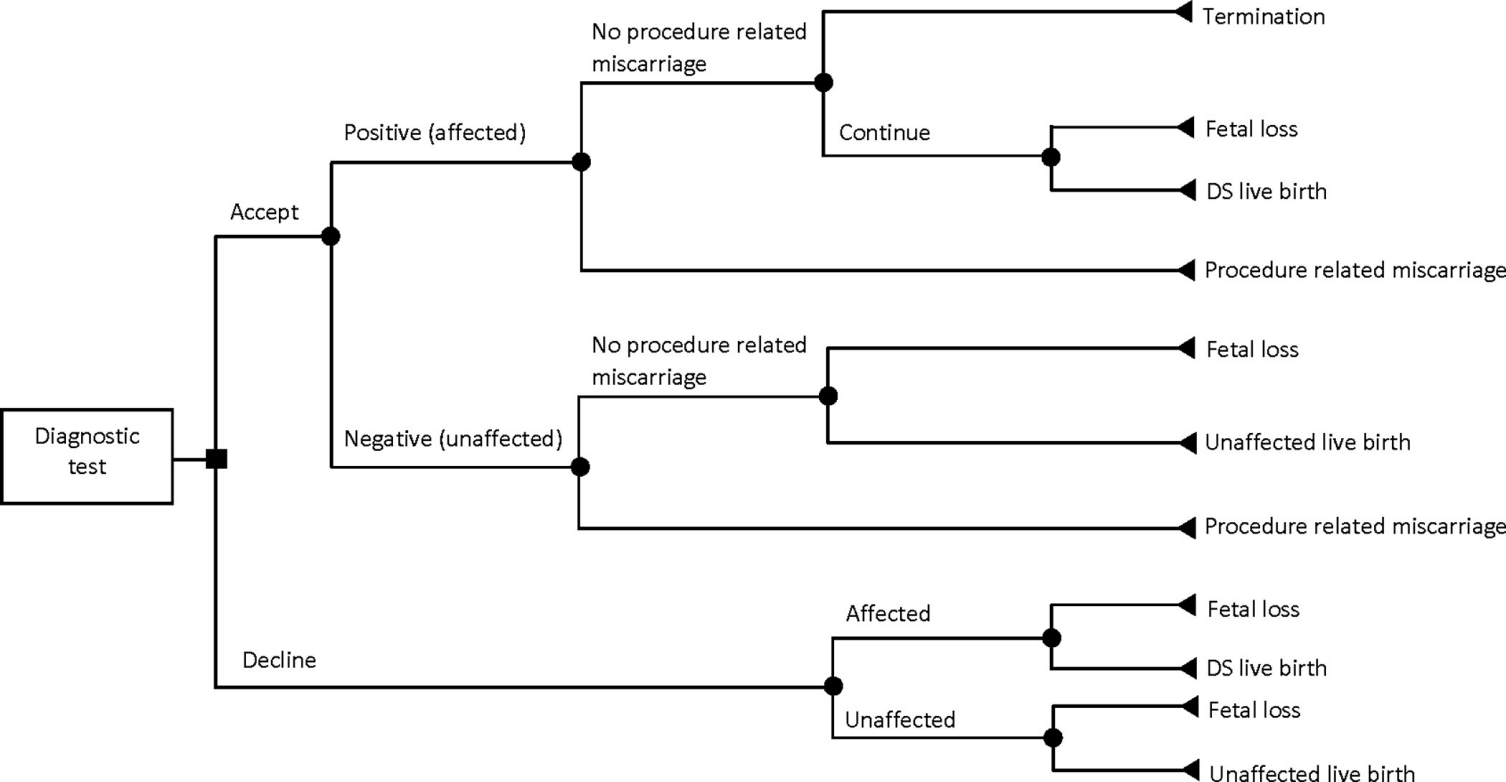
Pathway for diagnostic testing.

**Fig 3:**
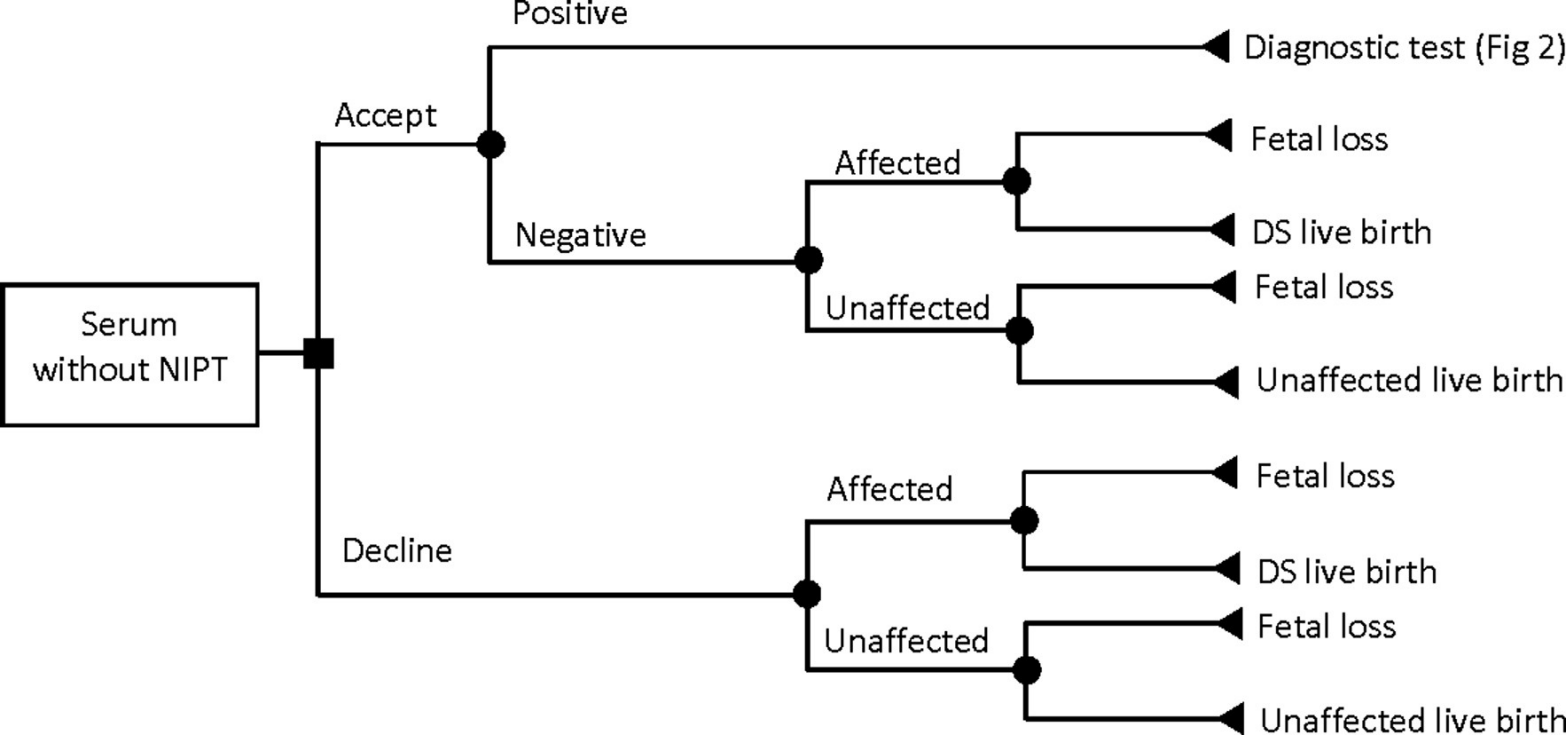
Pathway for serum-only screening strategy without non-invasive prenatal testing.

**Fig 4:**
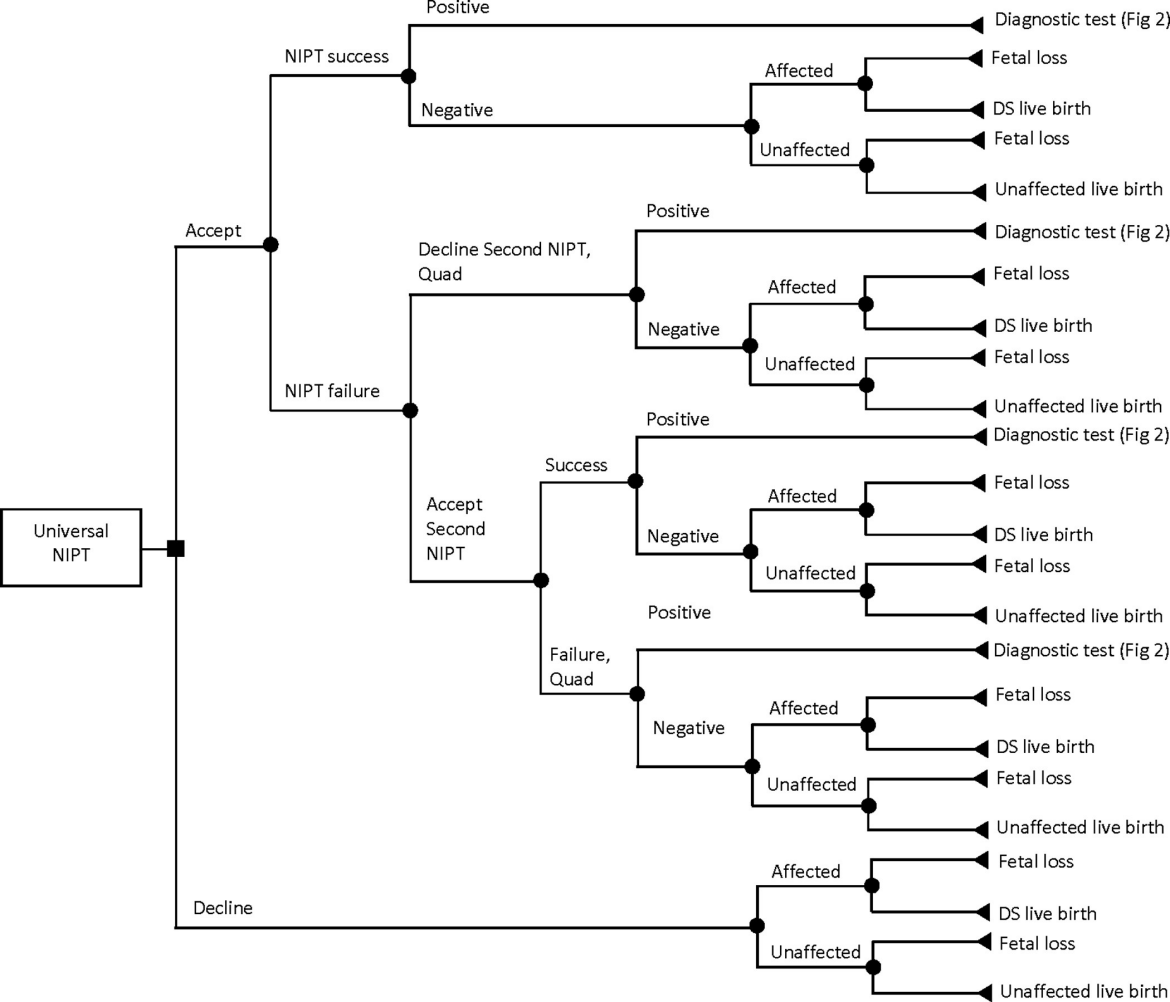
Pathway for universal non-invasive prenatal testing strategy.

In this study, we developed a microsimulation decision-analytic model to perform a CEA of prenatal screening and diagnostic strategies for DS. The target population consisted of singleton pregnant women representatives of the pregnant women population in BC, Canada. The model followed each simulated woman through the pregnancy pathway from the first decision to undergo (or decline) the prenatal screening tests to potential screening and diagnostic tests and pregnancy and post-pregnancy outcomes. The pathways in the model structure are detailed in Figs 1–4. It was assumed that a pregnant woman entered the model at the tenth week of pregnancy in all strategies.

The comparators were 1) contingent NIPT (i.e., standard screening with second-tier NIPT), 2) serum-only screening, and 3) universal NIPT (i.e., NIPT as a first-tier screening test). In the contingent NIPT strategy in BC (Fig 1), Serum Integrated Prenatal Screen (SIPS) is offered to all pregnant women as the first-tier screening test [18]. Women aged 35 and above have the option to undergo Integrated Prenatal Screen (IPS), which is SIPS in combination with NT ultrasound. Publicly funded NIPT is then offered to women with screening results indicating a high risk of a fetus with DS. Invasive diagnostic tests (CVS or amniocentesis) for fetal karyotyping are used to confirm diagnosis of DS among women with a positive NIPT result (Fig 2). Women with a positive initial screen also have the option of amniocentesis in BC. In our model, however, we have assumed that only women with a positive NIPT result would go onto invasive diagnostic testing. If the first NIPT is failed, a second NIPT test is offered to the pregnant women. Diagnostic testing is then offered if the second NIPT failed. For pregnant women with negative results from their first SIPS (or IPS) test or from the later publicly funded NIPT, we considered the probabilities of false negatives (affected fetus) and true negatives (unaffected fetus), as well as the respective probabilities of having fetal loss or live birth (Fig 1).

Serum-only screening was used as a base to compare with contingent NIPT. This strategy includes screening only with SIPS and IPS (Fig 3). Invasive diagnostic tests (CVS or amniocentesis) are performed for pregnant women with a positive screening result (Fig 2). For pregnant women with a negative screening result, we considered the probabilities of false negatives and true negatives as well as the respective probabilities of having fetal loss or live birth (Fig 3).

In universal NIPT, the SIPS and IPS are replaced by NIPT and it is assumed that publicly funded NIPT is offered to all pregnant women regardless of maternal age and pre-test risk of DS (Fig 4). Pregnant women with positive NIPT results are referred for invasive diagnostic testing (CVS or amniocentesis) for confirmation (Fig 2). It is assumed that after a failed NIPT, a second NIPT test is offered. After a second failed NIPT or if a second NIPT test is declined, a quad marker screen (Quad) is offered. Pregnant women with negative first or second NIPT results or negative Quad results may be true negatives or false negatives and may proceed to fetal loss or a live birth (Fig 4).

Pregnant women may choose to decline screening/testing at any stage. For them, we also have considered the probabilities of having an “affected” or “unaffected” fetus and the probabilities of having fetal loss or live birth. Moreover, although pregnant women over 40 years of age have the option to go directly to an invasive diagnostic test without previous screening, we have assumed that they would take the screening test before diagnosis due to its procedure-related risk of miscarriage [19]. Women with a confirmed case of carrying a fetus with DS are referred for counseling to be informed of their options to continue or terminate their pregnancy.

### Model inputs

#### Individual-specific inputs

For individual-specific parameters, we used existing data to estimate the relationship between model parameters and individual characteristics. In the model, the value of the individual-specific parameters is calculated for each simulated pregnant woman using these functions. The following is a brief description of the functions used in the model.

#### Maternal age distribution

A Gaussian model was used to simulate maternal age distribution [20]. The parameters of the Gaussian distribution were derived using age-specific fertility rates in BC and Canada and were then calibrated to reflect the distribution of age groups of pregnant women in BC.

#### Uptake rate of screening

In the model, we used the uptake of prenatal screening by maternal age from the BC Perinatal Health Report [21] and computed the probability of accepting the screening test for each simulated pregnant woman based on maternal age.

#### Probability of having a pregnancy with DS

For each woman entered into the model, the predicted risk of having a fetus with DS was derived from the maternal age-specific risk of having an affected live birth [22].

#### Risk of fetal loss for unaffected pregnancies

The age-specific risk of spontaneous fetal loss (including stillbirths and miscarriages) for unaffected pregnancies was derived from pregnancy outcomes statistics in BC [23]. It was then used to calculate individual-specific risk of miscarriage for each simulated woman through their pregnancy.

#### Population-level parameters

The value of population-level parameters that were the same for the sample are reported in Table 1. Parameters were obtained from published literature and from the BC perinatal database.

**Table 1:** Model inputs.

Parameter	Mean	Range	Distribution	References
***Probabilities***				
Risk of spontaneous fetal loss for DS affected pregnancy	36%	31%–43%	Beta (90, 160)	[25,27,28]
Uptake rate of diagnostic testing after positive serum screening	54%	43%–65%	Beta (44, 37)	[26]
Uptake rate of diagnostic testing after positive NIPT	80%	64%–96%	Beta (18, 5)	[26]
Failure rate of first NIPT	4%	1%–10%	Beta (3, 70)	[25,38]
Uptake rate of second NIPT after the failed first NIPT	83%	70%–100%	Beta (29, 6)	[39,25]
Failure rate of second NIPT	32.5%	10%–50%	Beta (7, 14)	[25]
Rate of pregnancy termination in case of aneuploidy diagnosis	90%	30%%–95%	Beta (19, 2)	[25]
Procedure-related fetal loss from diagnostic test	0.11%	0.05%–0.3%	Beta (7, 5955)	[19,25]
***Test Performances***				
SIPS: Sensitivity for DS by maternal age				
< 35 year	80%	85%-75%	Beta (50, 13)	[18]
35–39 year	86%	81%–91%	Beta (41, 7)	[18]
≥ 40 years	100%	95%–100%	Beta (8, 0.009)	[18]
SIPS: Specificity for DS by maternal age				
< 35 year	97%	94.5%–99.5%	Beta (44,1)	[18]
35–39 year	91%	88.5%–93.5%	Beta (118, 12)	[18]
≥ 40 years	80%	77.5%–825.5%	Beta (204, 5)	[18]
IPS: Sensitivity for DS by maternal age				
< 35 year	100%	98%–100%	Beta (9, 0.009)	[18]
35–39 year	95%	94%–96%	Beta (71, 4)	[18]
≥ 40 years	100%	98%–100%	Beta (9, 0.009)	[18]
IPS: Specificity for DS by maternal age				
< 35 year	97%	94.5%–99.5%	Beta (44, 1)	[18]
35–39 year	93%	90.5%–95.5%	Beta (96, 7)	[18]
≥ 40 years	83%	80.5%–85.5%	Beta (186, 38)	[18]
Quad: Sensitivity for DS by maternal age				
< 35 year	86%	81%–91%	Beta (41, 7)	[18]
35–39 year	85%	80%–90%	Beta (42, 7)	[18]
≥ 40 years	100%	95%–100%	Beta (9, 0.009)	[18]
Quad: Specificity for DS by maternal age				
< 35 year	96%	93.5%–98.5%	Beta (58, 2)	[18]
35–39 year	87%	84.5%–89.5%	Beta (157, 23)	[18]
≥ 40 years	69%	66.5%–71.5%	Beta (235, 106)	[18]
NIPT: Sensitivity for DS	99.2%	98.5%–99.6%	Beta (1019, 8)	[11,13,25]
NIPT: False positive rate for DS	0.3%	0.1%–0.5%	Beta (9, 2922)	[11,13,25]
***Costs***				
Cost of Consultation (Obstetrics/Gynecology)	$46.89		Fixed	[29]
Cost of Consultation (Medical Genetics)	$177		Fixed	[40]
Cost of SIPS (PAPP_A, AFP, uE3, hCG, and inhibin A)	$96.50		Fixed	Perinatal Services BC
Cost of Quad	$96.50		Fixed	Perinatal Services BC
Cost of nuchal translucency ultrasound	$124.03		Fixed	[29]
Cost of NIPT	$490		Fixed	Internal data
Cost of amniocentesis				
Transabdominal amniocentesis	$87		Fixed	[29]
Ultrasonic guidance for amniocentesis	$130		Fixed	[29]
Rapid aneuploidy detection	$157		Fixed	Perinatal Services BC
Cost of CVS	$727		Fixed	[30]
Cost of pregnancy termination	$1717	$1284-$2146	Gamma (16; 0.009)	CIHI Data
Cost of miscarriage (fetal loss)	$713	$535-$891	Gamma (16; 0.022)	[33]
Cost of DS*	$127,256	$121,532-$132,980	Gamma (494; 0.004)	[31]
***Utilities***				
Screening test with low-risk results	0.931	0.777–1	Beta (1.59; 0.118)	[17]
Diagnostic testing with normal results	0.921	0.76–1	Beta (1.66; 0.143)	[17]
Fetal loss, child 2 years later	0.88	0.702–1	Beta (2.052; 0.280)	[17]
Pregnancy loss; no future pregnancy	0.590	0.277–0.903	Beta (0.867; 0.602)	[17]
Pregnancy termination after positive diagnostic test	0.771	0.503–1	Beta (1.124; 0.334)	[17]
Child with DS or another intellectual disability	0.480	0.175–0.785	Beta (0.808; 0.875)	[17]

DS, Down syndrome; NIPT, non-invasive prenatal testing; SIPS, serum integrated prenatal screen; IPS, integrated prenatal screen; PAPP A, pregnancy-associated plasma protein A; AFP, alpha-fetoprotein; uE3, unconjugated estriol; hCG, human chorionic gonadotropin; CVS, chorionic villus sampling; CIHI, Canadian Institute for Health Information.

* Incremental health care cost of a child with DS.

#### Number of pregnant women

We estimated the number of pregnant women undergoing prenatal screening in BC using the live births and fetal deaths data from Statistics Canada and considered that 91% of abortions were done before week 12 of pregnancy [24].

#### Sensitivity and specificity of serum screening and NIPT

Detection rate and false positive rate of serum screening vary by maternal age. As such, sensitivity and specificity of SIPS and IPS for different maternal age groups were obtained from the British Columbia Perinatal Data Registry (Perinatal Services BC) [18]. The NIPT sensitivity and specificity values used in our model were obtained from a previous CEA study based on meta-analysis results [11,13,25].

#### Uptake rate of diagnostic test after positive screening

We used different mean of uptake rate of diagnostic testing after serum screening (54%) and NIPT (80%) [26].

#### NIPT success rate

We assumed that the first NIPT mean of failure rate is 4% and 32.5% for the second NIPT [25].

#### Risk of fetal loss for affected pregnancies

The risk of spontaneous fetal loss for DS affected pregnancies applied to pregnant women who were false negative cases, those who declined screening/testing at any stage, or chose to continue pregnancies with positive diagnostic testing results. Their risk of fetal loss was usually higher than the risk of fetal loss for unaffected pregnancies. The risk estimate we used was 36% [25,27,28].

#### Unit costs

The majority of the unit costs used in the model (Table 1) were based on the BC Medical Services Plan Payment Schedules [29,30].

#### Cost of DS

We conducted a search of the literature to find an estimate of the incremental health care cost for children with DS compared to typically developing children in Canada but no studies were found. As such, we referenced a recent study by Kageleiry et al. [31] using United States (US) data, which estimated the excess health care costs of children with DS compared to typically developing children aged from birth to 18 years. The costs included medical costs (i.e., inpatient, outpatient, emergency room, and home health agency) and pharmacy costs. To be consistent with the Canadian public health care system in which all of these health care expenses would be publicly paid for, we considered the sum of the incremental out-of-pocket health care co-payment for children with DS and the incremental total health care expenditures for private third-party insurers by different age groups. To address the health care cost difference between Canada and US due to different health care systems, we first converted the incremental cost estimates in the US study by Kageleiry et al. [31] (in present values in 2013) to incremental cost estimates in Canada using the ratio between health expenditure per person in the US and that in Canada [32]. The ratio was estimated from the study by Lorenzoni et al. [32], who reported the health expenditure per person in US$ in both US (US$7,212) and Canada (US$3,796). We then converted the incremental cost estimates in US$ to CAN$ using purchasing power parity.

#### Cost of fetal loss

We used an estimate from a study that evaluated the cost of unintended pregnancies in Canada [33] and inflated the cost based on the national health expenditure per capita over years in Canada [32].

#### Cost of NIPT

The average of different prices for NIPT available commercially from internal data was used in the base case.

#### Utility weights

For the base case, we used women’s utility weights from a US study which estimated time trade-off utilities for short and long-term outcomes of prenatal testing strategies [17]. These included utilities for screening tests with low-risk results, diagnostic tests with normal results, pregnancy termination after positive diagnostic test results, and having a child with DS. In addition, according to the results of the previous studies [17,34,35], utility of pregnancy loss is dependent on whether a future birth occurs after the fetal loss. As such, we estimated the probability of a future birth in two years after miscarriage for each simulated woman based on the maternal age (i.e., an individual-specific parameter) [36,37] and used this probability to calculate the utility of fetal loss for that specific woman. In the model, we used utility decrement for all health states.

### Validation and calibration

We validated the model structure by using data from BC and Canada, [21,41,42]. The model-generated results of unaffected live births, DS births, and fetal loss were compared to the observed values. Calibration was performed to the model inputs to produce a close fit to the setting-specific data.

### Analyses

To address uncertainty around the model parameters, the expected values of costs and QALYs in the base case and all scenario analysis were obtained through probabilistic analysis from a Monte Carlo simulation. All analyses were conducted by repeating the micro-simulation 1,000 times for the sample of 45,605 pregnant women in BC. The costs in the model included costs until the end of the pregnancy and incremental long-term costs (18 years after pregnancy). All costs were reported in 2017 Canadian dollars. Effectiveness was measured in terms of change in QALY loss for pregnant women during the same time period as costs. A 3% discount rate was used.

In addition to the base case, we conducted a series of scenario analyses to quantify the influence of changes in input parameters and model assumptions on outcomes. As well, a threshold analysis was performed to assess the impact of NIPT cost on the outcomes and to identify the threshold level that would make universal NIPT a cost-effective strategy.

## Results

### Base case analysis

**Fig 5:**
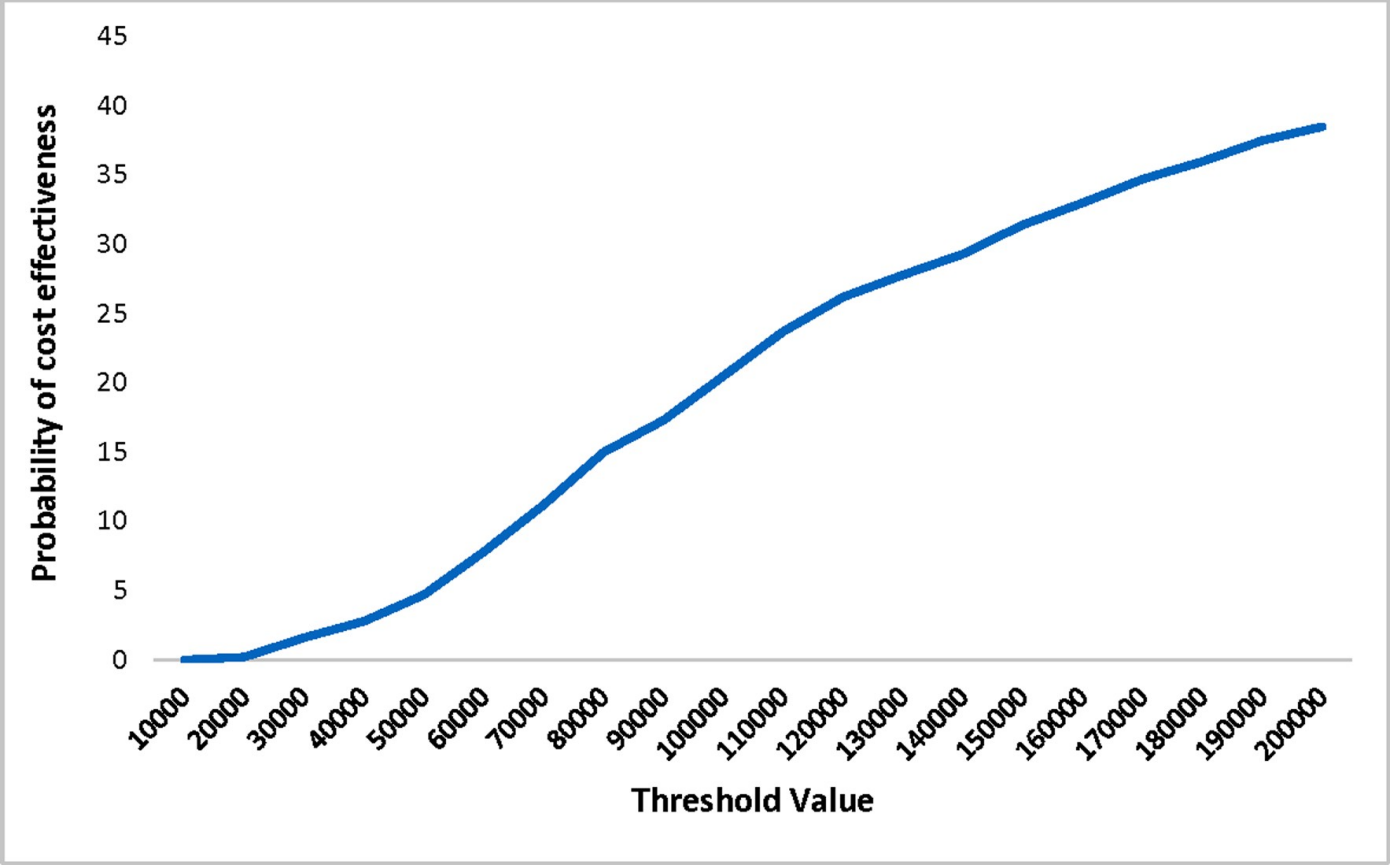
Cost-effectiveness acceptability curve–base case.

Results of the sequential CEA are presented in Table 2. Contingent NIPT dominated over the serum-only strategy with lower long-term cost, lower cost per case detected, and slightly higher QALY gain (or lower QALY loss). Comparison between universal and contingent NIPT strategies showed that the long-term cost of universal NIPT per pregnant woman was $507 compared to $314 for contingent NIPT and resulted in an incremental cost of $193 (Table 3). Using NIPT as a first-tier screening test yielded a small gain in QALY (0.0925 QALY loss in contingent NIPT versus 0.0918 in universal NIPT). But as seen in the cost-effectiveness acceptability curve in Fig 5, at the willingness-to-pay (WTP) threshold of $50,000/ QALY gain, universal NIPT was only cost-effective in 5% of the runs as compared to contingent NIPT. Universal NIPT resulted in more detected DS cases compared to contingent NIPT (on average 117 versus 108 for universal and contingent NIPT, respectively). Considering cost until the end of pregnancy, costs per detected case were $124,076 versus $47, 210 for universal and contingent NIPT, respectively.

**Table 2:** Base case results at population level.*

Strategy	Mean of DS cases detected	Mean of costs until the end of the pregnancy ($)	Mean of cost per case detected ($)	Mean of long-term costs ($)	Mean of QALY loss	Sequential incremental cost per QALY gained
Serum-only	74	4,869,625	67,395	16,549,322	4363	
Contingent NIPT	108	5,082,323	47,210	14,314,449	4221	Dominant
Universal NIPT	117	14,555,015	124,076	23,125,234	4188	267,103

DS, Down syndrome; QALY, quality-adjusted life year; NIPT, non-invasive prenatal testing.

*Results among a sample of 45,605 pregnant women.

**Table 3:** Results of scenario analyses.

	Contingent NIPT		Universal NIPT				
Scenario	Cost $ (CI)	QALY loss (CI)	Cost $ (CI)	QALY loss (CI)	Incremental Cost (CI)	Incremental QALY (CI)	Probability of universal NIPT being cost-effective*
**Base case**	314 (242, 386)	0.0925 (-0.2012, 0.3864)	507 (437, 577)	0.0918 (-0.2014, 0.3851)	193 (150, 236)	0.0007 (-0.0023, 0.0038)	4.7%
NIPT cost = $400	311 (240, 382)	0.0925 (-0.2012, 0.3864)	453 (383, 523)	0.0918 (-0.2014, 0.3851)	142 (99, 185)	0.0007 (-0.0023, 0.0038)	11.4%
NIPT cost = $300	308 (237, 379)	0.0925 (-0.2012, 0.3864)	393 (323, 463)	0.0918 (-0.2014, 0.3851)	85 (42, 127)	0.0007 (-0.0023, 0.0038)	26.7%
NIPT cost = $200	305 (234, 375)	0.0925 (-0.2012, 0.3864)	332 (262, 402)	0.0918 (-0.2014, 0.3851)	28 (-14, 70)	0.0007 (-0.0023, 0.0038)	50.6%
NIPT cost = $150	303 (232, 373)	0.0925 (-0.2012, 0.3864)	302 (232, 372)	0.0918 (-0.2014, 0.3851)	-1 (-43, 41)	0.0007 (-0.0023, 0.0038)	66.3% (Universal NIPT is dominant 46.7%)
Mean of incremental direct medical cost of DS = $152,707	354 (269, 440)	0.0925 (-0.2012, 0.3864)	545 (461, 629)	0.0918 (-0.2014, 0.3851)	190 (139, 242)	0.0007 (-0.0023, 0.0038)	5.4%
Mean of incremental direct medical cost of DS = $101,805	274 (215, 332)	0.0925 (-0.2012, 0.3864)	469 (413, 526)	0.0918 (-0.2014, 0.3851)	196 (157, 235)	0.0007 (-0.0023, 0.0038)	4.3%
Mean of uptake of diagnostic test after NIPT & serum = 54%	369 (298, 440)	0.1002 (-0.2132, 0.4138)	568 (498, 638)	0.0998 (-0.2132, 0.4128)	199 (144, 253)	0.00004 (-0.0031, 0.0040)	6.6%
Mean of uptake of diagnostic test after NIPT & serum = 80%	315 (242, 387)	0.916 (-0.1993, 0.3825)	509 (439, 579)	0.0910 (-0.1993, 0.3812)	194 (152, 236)	0.0006 (-0.0024, 0.0037)	5.1%
Mean of termination rate = 0.31	426 (354, 498)	0.0908 (-0.1886, 0.3702)	629 (561, 697)	0.0907 (-0.1882, 0.3696)	203 (140, 266)	0.0001 (-0.0040, 0.0042)	5.8%
Mean of termination rate = 0.70	351 (278, 424)	0.0943 (-0.2087, 0.3974	548 (477, 619)	0.9038 (-0.2085, 0.3961)	197 (145, 248)	0.0005 (-0.0027, 0.0038)	6.3%
Mean of termination rate = 0.95	304 (230, 378)	0.0863 (-0.2073, 0.3798)	497 (422, 572)	0.0856 (-0.2076, 0.3787)	193 (152, 234)	0.0007 (-0.0022, 0.0036)	4.7%

NIPT, non-invasive prenatal testing; CI, confidence interval; QALY, quality-adjusted life year; DS, Down syndrome.

*At a threshold of $50,000/QALY gained.

### Scenario analyses

#### Threshold analysis of NIPT cost

**Fig 6:**
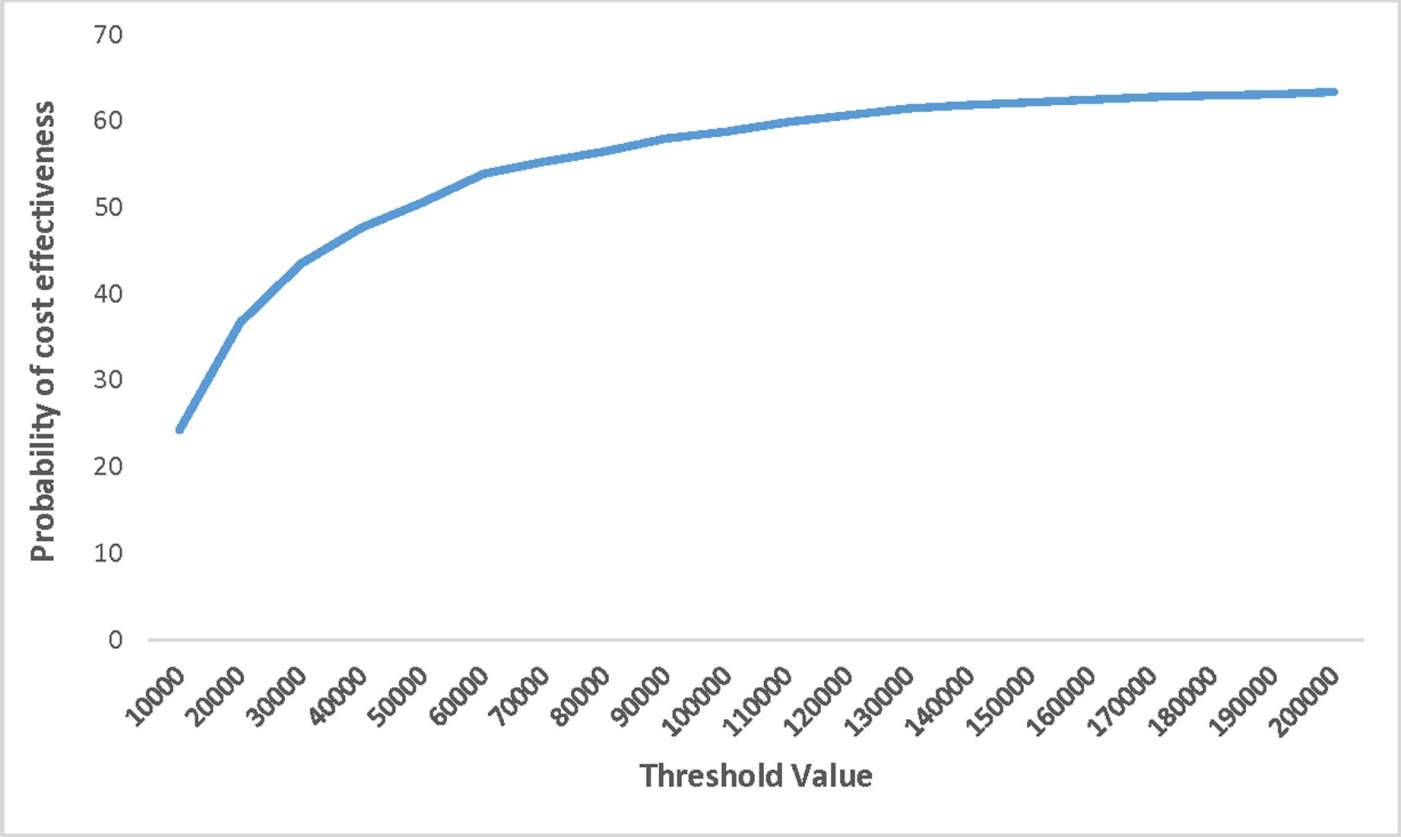
Cost-effectiveness acceptability curve–cost of non-invasive prenatal testing at $200.

We tested the effects of changes in NIPT cost on the outcome of the model by running a series of scenario analyses. The model was run for cost of NIPT at $400, $300, $200, and $150. With a NIPT cost of $400 and $300, at a WTP of $50,000 per QALY, the probability that universal NIPT is cost-effective was 11% and 27%, respectively (Table 3). If the cost of NIPT drops to $200, the incremental long-term cost of universal versus contingent NIPT would be $28 per pregnant women. Under this scenario, the probability of universal NIPT being a cost-effective strategy at $50,000 per QALY threshold increased to 51% (Fig 6). Also, the cost per case detected became $43,241 and $56,137 for contingent and universal NIPT, respectively.

#### Incremental direct medical cost of DS

In the base case, we used an estimate of $127,256 for the incremental direct medical cost of DS (calculated based on the results of Kageleiry et.al [31]). We tested the sensitivity of the outcomes to this cost by conducting two other scenario analyses with the cost of $152,707 and $101,805. As the results show, the outcome of the model is not changed when the incremental direct medical cost of DS is varied over this range.

#### Uptake rate of diagnostic test

In the base case, uptake rate of the diagnostic test after positive serum screening was 0.54 and after positive NIPT was 0.8 [26]. In scenario analyses, we ran different scenarios to test the impact of the assumption of same uptake of diagnostic test after serum screening and NIPT. The results showed that the assumptions regarding uptake of the diagnostic test had no impact on the model outcomes (Table 3).

#### DS pregnancy termination rate

To assess the effect of changes in the pregnancy termination rate on the outcome of the model, we ran the model with different mean of DS pregnancy termination rates at 30%, 70%, and 95%. With assumed termination rate of 30%, costs per pregnant woman for both scenarios and the incremental cost were higher compared to the base case. The probability of universal NIPT being cost-effective compared to contingent NIPT was 5.8%. The results of the scenario with termination rate at 70% showed increased cost per pregnant woman for both strategies compared to the base case value but incremental cost was almost unchanged ($193 in the base case versus $197 in the scenario with 70% termination rate). At a willingness-to-pay of $50,000 per QALY, universal NIPT had only 6% probability of being cost-effective compared to contingent NIPT.

Under the scenario that DS pregnancy termination rate was 95%, incremental cost per pregnant woman was $222 and incremental QALY gain was 0.0007. At the willingness-to-pay threshold of $50,000/QALY, there was only a 5% probability that universal NIPT was cost-effective compared to contingent NIPT. The results of these scenarios showed that the outcome of the model is not affected by the value of the termination rate.

## Discussion

Using a microsimulation model, we conducted a CEA of prenatal screening and diagnostic strategies for DS from the BC publicly-funded health care payer’s perspective. We found that at the current price of NIPT paid through the BC publicly funded program, the contingent NIPT strategy dominated over the serum-only strategy while the universal NIPT strategy was not cost-effective based on a WTP threshold of $50,000/QALY as applied in Canada. Our cost-effectiveness results were highly sensitive to the price of NIPT. Universal NIPT could become cost-effective in comparison to the contingent NIPT if the NIPT price was set at $200 or lower.

Our results are consistent with four previously published CEAs (one in the UK [26] and three in the US [43–45]), whereby contingent NIPT dominated the conventional screening strategy [26,43–45]. However, two CEAs reported opposite findings where contingent NIPT is dominated by the conventional strategy: first-trimester screening (FTS) (screening that combines maternal age with maternal serum markers and NT ultrasound) when the risk cut-off is at or below 1/500 [46] or when the same test uptake is assumed for contingent NIPT and FTS [47]. Using QALY as an effectiveness measure, Kaimal et al., found that the conventional strategy was the optimal strategy for most women in the US [34].

In addition, our finding that universal NIPT was more effective but more costly than the conventional screening strategy is consistent with all previous CEAs except for three conducted in the US. The two CEA studies by Walker et al. demonstrated that universal NIPT dominated over the conventional screening strategy [45,48] as well as contingent NIPT [45] when taking a societal perspective, which included incremental direct medical costs, incremental education costs, and indirect costs of lost productivity due to morbidity and mortality associated with DS. However, when they took a government perspective by excluding indirect costs, they found that universal NIPT was not cost-effective [45,48] and contingent NIPT dominated over the conventional screening strategy [45], which is consistent with our findings. Fairbrother et al. concluded that universal NIPT dominated over FTS when NIPT costs were US$453 or less [49].

The two published Canadian CEA studies showed different findings: contingent NIPT was more costly but more effective than the conventional screening strategy in an Ontario study [50], whereas it was less costly but less effective than strategies without NIPT in a Quebec study [25]. However, the reported cost-effectiveness outcomes in terms of the incremental cost per additional DS case detected were not comparable between their studies and ours due to different conventional screening strategies compared, costs of conventional screening strategies, costs of NIPT ($795 versus $490 applied in our CEA), and decision analytic models employed [25,50].

### Strengths and limitations

Our study is one of the few CEAs that have reported QALY as the effectiveness measure. Since almost all previous CEAs reported ICER as $ per case detected, it is difficult to ascertain whether contingent NIPT or universal NIPT is cost-effective unless they are dominant or dominated. To increase the comparability in CEAs across different diseases and interventions to help decision makers make funding decisions, QALYs are recommended as the effectiveness measure according to guidelines for conducting CEAs and the WTP threshold is commonly set as $/QALY (e.g., $50,000/QALY applied in Canada) [51,52]. Our study findings allow for this comparability. However, CEA findings are only one piece of evidence that decision makers may consider in their funding decisions. In addition, they often consider clinical effectiveness, ethics, implementation complexity, and other criteria [53,54].

By employing a microsimulation model instead of a cohort-based model, we accomodated the heterogeneity among pregnant women and were able to track their individual pathways. Furthermore, the information we used, such as maternal age distribution, maternal age-specific update rate of screening, and costs of health care services were obtained from the most recent data sources as well as real-world observations.

In terms of study limitations, we did not take into account other chromosomal abnormalities such as Edward’s syndrome (trisomy 18) or trisomy 13. Thus, our analysis may have underestimated the potential benefit of NIPT. However, since these abnormalities are relatively rare, we believe the impact is minimal. Secondly, our estimate of the incremental health care cost of children with DS compared with typically-developing children was based on US data as no Canadian data was available. We stringently converted the estimate into Canadian dollars using the ratio of health care expenditure between US and Canada. We also considered the range of the cost estimate in scenario analyses to capture the impact of this parameter on the outcomes. Lastly, CEAs require a specific perspective and since we took the BC publicly funded health care payer’s perspective, our target population in the current study was pregnant women in BC, Canada. However, the modeling framework used in this study is not BC-specific and can be adjusted to evaluate cost and effectiveness of prenatal screening and diagnostic strategies in various settings.

## Conclusion

Including NIPT in prenatal screening programs for DS was shown to be beneficial over conventional testing. At current prices of NIPT, implementation of NIPT as a second-tier screening test is more cost-effective than deploying it as a first-tier screening test. However, a further decrease in the price of NIPT could make first-tier NIPT screening more cost-effective.
